# Alveolar Ridge Split Technique With Simultaneous Implant Placement: A Clinical Case Series With One-Year Follow-Up

**DOI:** 10.7759/cureus.115750

**Published:** 2026-09-03

**Authors:** Anthony Challita, Fady Abillamaa, Elie Maalouf

**Affiliations:** 1 Periodontology, Lebanese University Faculty of Dental Medicine, Beirut, LBN

**Keywords:** alveolar ridge augmentation, alveolar ridge split, dental implant placement, guided bone regeneration (gbr), simultaneous implant placement and bone regeneration

## Abstract

Ridge resorption following tooth loss may limit ideal three-dimensional implant placement. The alveolar ridge split technique (ARST) is used for horizontal ridge augmentation and may permit simultaneous implant placement in selected narrow ridges. This case series aimed to assess horizontal bone gain following ARST with simultaneous implant placement and evaluate short-term ridge stability one year after loading.

Three systemically healthy patients presenting with maxillary horizontal bone width of less than 6 mm were included. All underwent ARST using piezosurgery and chisels with simultaneous implant placement, followed by xenogeneic bone grafting and coverage with a pericardium membrane stabilized using titanium pins. Second-stage surgery was performed four months postoperatively, and definitive crowns were delivered one month later. Cone beam computed tomography (CBCT) and clinical measurements of ridge width were obtained preoperatively, immediately postoperatively, at crown delivery, and one year after functional loading.

All three cases healed uneventfully, and all implants remained in function at one year after loading. The mean radiographic horizontal bone gain was 3.83 mm (range, 2.9-4.8 mm), while the mean clinical gain was 4.16 mm. A minimum buccal bone thickness of 2 mm was maintained around all implants at the one-year follow-up. No implant loss, membrane exposure, or major postoperative complications were observed.

Within the limitations of this three-patient case series and one-year follow-up, ARST with simultaneous implant placement and guided bone regeneration was feasible and associated with favorable short-term clinical and radiographic outcomes in carefully selected narrow maxillary ridges. The technique achieved horizontal ridge augmentation and maintenance of adequate buccal bone thickness at one year after loading in all cases. However, these findings should not be interpreted as evidence of broad treatment predictability, comparative effectiveness, or long-term stability. Larger prospective comparative studies with longer follow-up are needed to confirm these preliminary findings and refine case-selection criteria.

## Introduction

Implant dentistry is an established treatment modality for replacing missing teeth, ranging from single-tooth restorations to full-mouth rehabilitation [1]. Dental implants demonstrate survival rates exceeding 90% when placed in adequate bone volume and under proper prosthetic planning [1]. However, following tooth extraction, the alveolar ridge undergoes resorption in the vertical, transverse, and sagittal dimensions, with the most pronounced loss typically occurring in the transverse plane during the first three months, particularly in the mandible [2,3]. Horizontal bone loss has been reported to range between 3.8 and 6.1 mm within 3-12 months after extraction, and ridge width may be reduced by up to 50% of its original value [4,5]. These changes can compromise ideal three-dimensional implant positioning and the maintenance of at least 1.5 mm of bone around the implant [3].

Implant placement requires adequate residual bone height and width, and a ridge width of less than 6 mm is generally considered narrow and often necessitates horizontal augmentation before implant placement [2,6]. The alveolar ridge is the portion of the jawbone that surrounds and supports the teeth. Following tooth loss, this bone commonly becomes narrower, particularly on its outer (buccal) aspect, which may leave insufficient bone to place an implant in an ideal three-dimensional position. Horizontal ridge augmentation comprises surgical procedures intended to increase ridge width and provide adequate bone support around an implant. In this context, the ridge split technique has been reported as a treatment option for horizontal ridge augmentation, with previous clinical reports describing high implant survival rates in appropriately selected sites [3].

First introduced by Tatum in the 1970s and subsequently modified by Summers and Simion, the alveolar ridge split technique (ARST) involves creating a controlled cut along the crest of the alveolar ridge (osteotomy) and gently expanding the ridge. This produces a controlled, incomplete fracture, often termed a greenstick fracture, that separates the outer buccal cortical plate from the inner palatal or lingual cortical plate while maintaining soft-tissue attachment and blood supply [3,7-9].

The ARST procedure requires specific anatomical conditions to be successful: a minimum horizontal width of 2 mm with cancellous bone between cortical plates, a minimum residual bone height of 10 mm, absence of concavity, and maintenance of at least 1 mm safety distance from adjacent teeth [4,9]. When properly indicated, this technique offers several advantages, including simultaneous ridge augmentation and implant placement, elimination of the need for a donor site, reduced risk of membrane and graft exposure, and shortened treatment time and morbidity [3,7]. However, limitations include increased risk of buccal plate fracture, particularly in the mandible, potential lack of primary implant stability, and compromised implant positioning in certain clinical scenarios [10-12].

The indication for ARST differs according to the anatomical characteristics of the maxilla and mandible. The maxilla generally presents more favorable bone elasticity and a lower cortical bone density, which may facilitate controlled ridge expansion. In contrast, the denser cortical structure of the mandible may limit expansion and increase the risk of buccal cortical plate fracture. Therefore, the present case series was restricted to maxillary sites with adequate residual vertical bone height and a narrow horizontal ridge, and its findings should not be generalized to mandibular ridge deficiencies [4,10-12].

Although ARST with simultaneous implant placement has been described in previous case reports and clinical series, further documentation using standardized serial clinical and cone-beam computed tomography (CBCT) measurements is needed to characterize dimensional changes after treatment and during functional loading. Therefore, ARST may offer a practical approach for patients with narrow maxillary ridges by allowing horizontal ridge augmentation and implant placement during the same surgical procedure.

The present three-patient case series adds to the available evidence by reporting a uniform maxillary treatment protocol combining ARST, simultaneous implant placement, xenogeneic bone grafting, and pericardium membrane coverage. Ridge width was assessed clinically and radiographically before surgery, immediately after surgery, at crown delivery, and one year after functional loading. The aim of this case series was to evaluate horizontal bone gain and the short-term clinical and radiographic stability of the augmented ridge in narrow maxillary sites.

## Case presentation

Patient selection

Patients requiring implant placement in the maxilla and presenting with a horizontally deficient alveolar ridge were screened for eligibility. Eligible patients presented with a maxillary edentulous site requiring implant-supported rehabilitation and an inadequate horizontal ridge dimension for ideal three-dimensional implant placement. Horizontal ridge deficiency was confirmed clinically and by CBCT, with a ridge width of less than 6 mm. Inclusion criteria were systemic health, no known drug allergies, and light smoking status (≤5 cigarettes/day). Exclusion criteria included uncontrolled systemic disease, heavy smoking, early implant placement (4-8 weeks after tooth extraction), the presence or history of untreated periodontal disease, the presence or history of oral potentially malignant disorders or premalignant lesions, and previous radiotherapy to the head and neck region. A comprehensive preoperative intraoral examination was performed for all patients, and no suspicious oral mucosal lesions were identified. Three patients fulfilled these criteria and were enrolled after receiving detailed information about the procedure and providing written informed consent.

Clinical protocol

Before surgery, all patients received initial periodontal therapy, including scaling and individualized oral hygiene instructions. Probing pocket depth and bleeding on probing were recorded at baseline, and clinical and radiographic examinations were performed. A diagnostic wax-up and mock-up were used to guide prosthetically driven implant positioning.

The standardized treatment protocol consisted of ridge split procedure; simultaneous implant placement; guided bone regeneration using Cerabone (Botiss, Germany) and a Jason pericardium membrane (Botiss, Germany), stabilized with titanium pins (Botiss, Germany); second-stage surgery 16 weeks after the initial procedure; impression taking one month after second-stage surgery; delivery of the definitive crown; clinical ridge-width measurements using a North Carolina periodontal probe before surgery, immediately after surgery, and at second-stage surgery; and CBCT imaging before surgery, immediately after surgery, at definitive crown delivery, and one year after functional loading.

Clinical ridge-width measurements were obtained before surgery, immediately after surgery, and at second-stage surgery, when direct access to the ridge permitted clinical assessment. No direct clinical ridge-width measurement was obtained at the one-year post-loading visit to avoid invasive manipulation of the healed peri-implant tissues. CBCT measurements were obtained at all four radiographic time points to assess dimensional changes of the augmented ridge noninvasively during functional follow-up.

Presurgical examination

A comprehensive clinical and radiographic assessment was performed for each patient. The clinical presentation consisted of a healed maxillary edentulous site requiring implant-supported rehabilitation, with insufficient horizontal ridge width for prosthetically driven implant placement. CBCT was used to confirm ridge width, evaluate residual vertical bone height, identify the relationship with adjacent teeth and anatomical structures, and assess the presence of adequate cancellous bone between the cortical plates.

Horizontal ridge width was assessed as the buccolingual distance between the outer surfaces of the buccal and palatal cortical plates at the planned implant site. On CBCT images, measurements were obtained at the corresponding anatomical implant site on cross-sectional images oriented perpendicular to the alveolar crest. Clinical ridge width was measured at the corresponding implant site using a North Carolina periodontal probe positioned perpendicular to the long axis of the alveolar ridge. All measurements were recorded in millimeters by the same calibrated examiner (A.C.).

Only patients with good oral hygiene, adequate vertical bone height, and clinical and radiographic features compatible with ARST were scheduled for surgery.

Surgical procedure

Under local anesthesia (Dilocain 2%), a full-thickness mucoperiosteal flap was elevated following intrasulcular incisions on the teeth adjacent to the edentulous area and a midcrestal incision over the defect (Figure 1).

**Figure 1:**
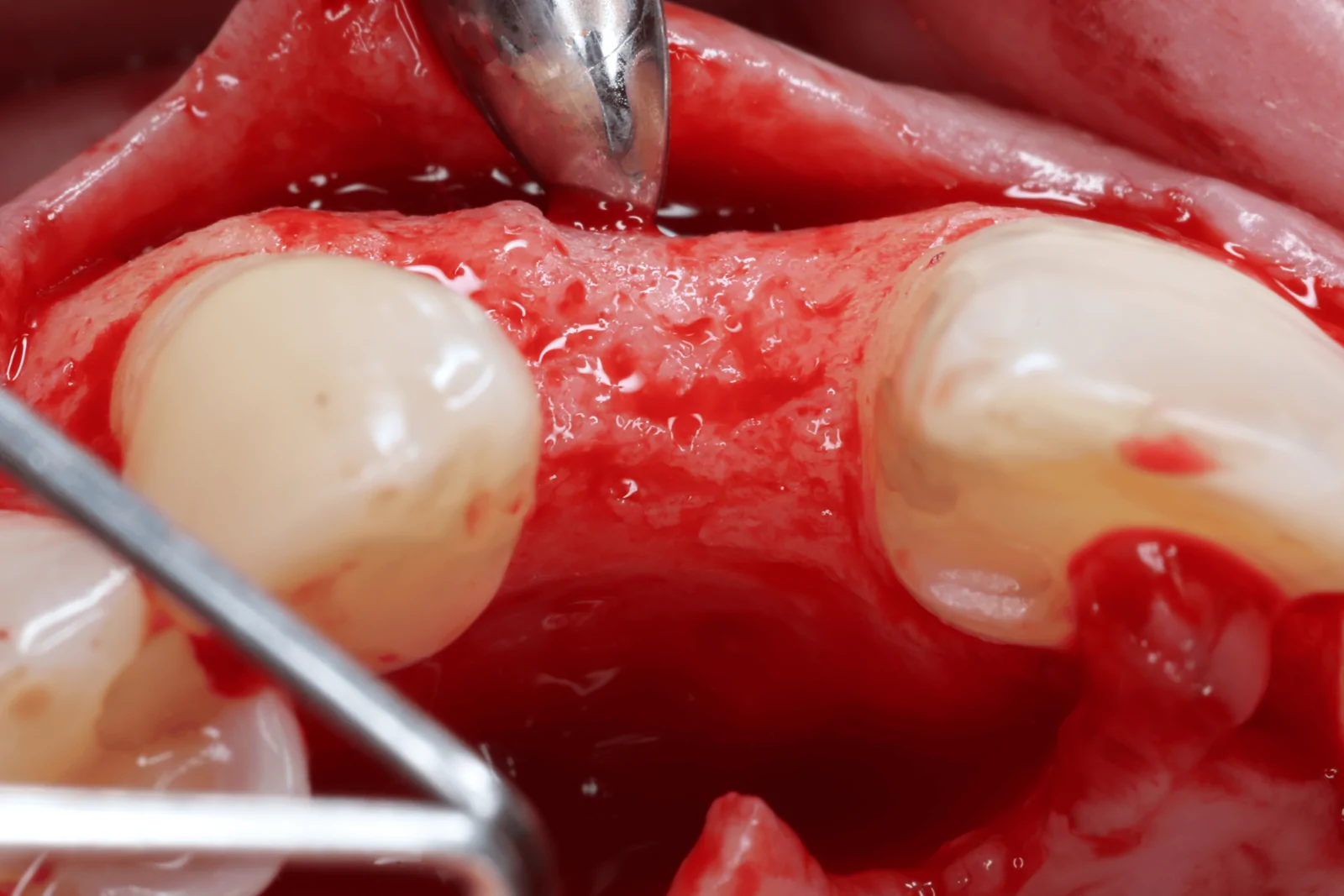
Preoperative clinical view of the maxillary edentulous ridge showing horizontal ridge deficiency at the planned implant site.

Horizontal and vertical osteotomies were performed at the edentulous site using a piezosurgical device (Mectron) while maintaining at least 1 mm of safety distance from adjacent teeth (Figure 2).

**Figure 2:**
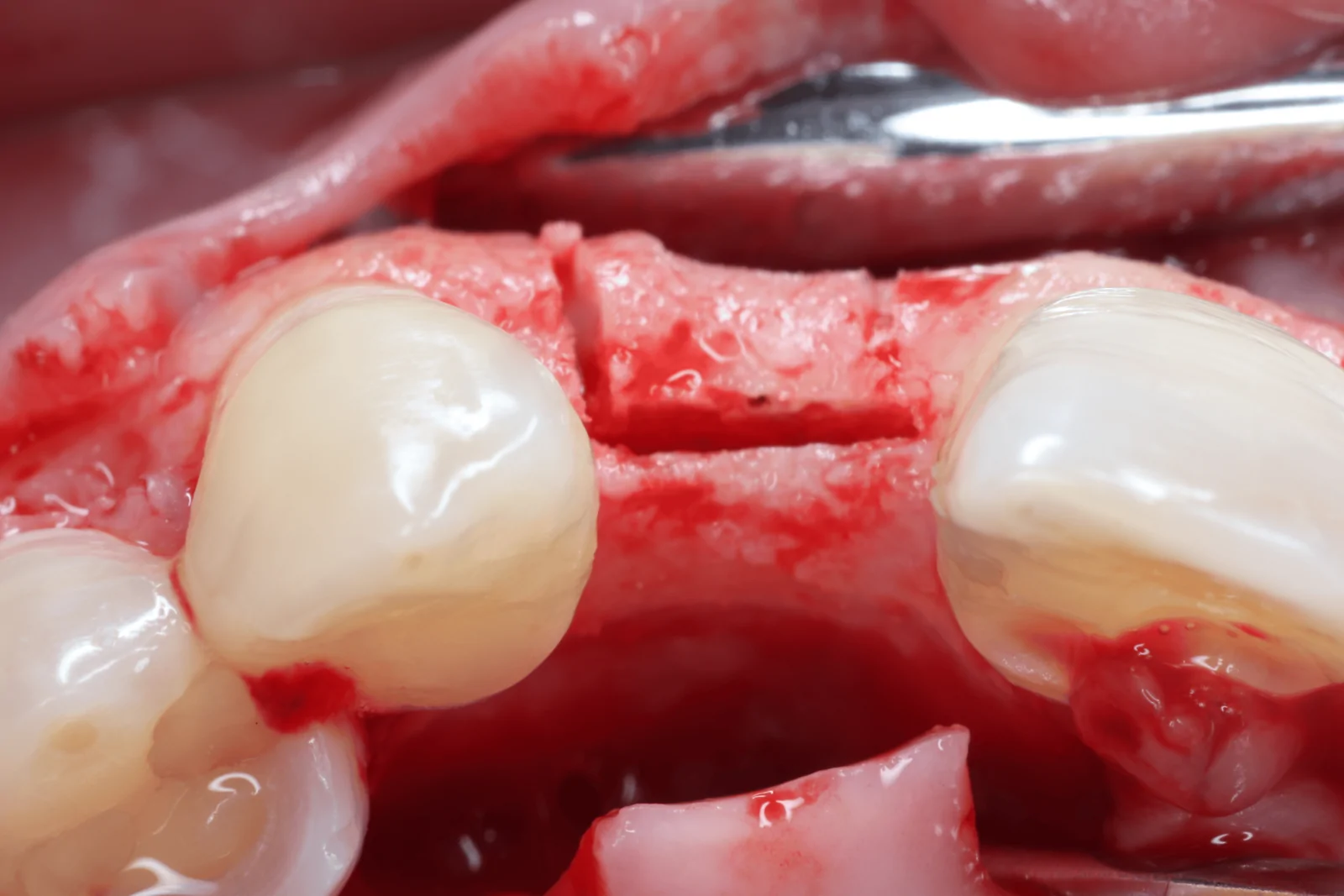
Intraoperative view of the alveolar ridge split procedure. Horizontal crestal and vertical releasing osteotomies were performed using piezosurgery to permit controlled mobilization of the buccal cortical plate.

These osteotomies created a controlled greenstick fracture that allowed buccal plate mobilization.

Sequential chisels of increasing thickness were then used to gradually expand the ridge and maximize buccal plate mobility. Implant osteotomies were prepared according to the manufacturer's protocol for the Klockner implant system, with drilling speed kept below 400 rpm to enhance primary stability and reduce the risk of buccal plate fracture. Implants were placed in the planned three-dimensional position and covered with cover screws (Figure 3).

**Figure 3:**
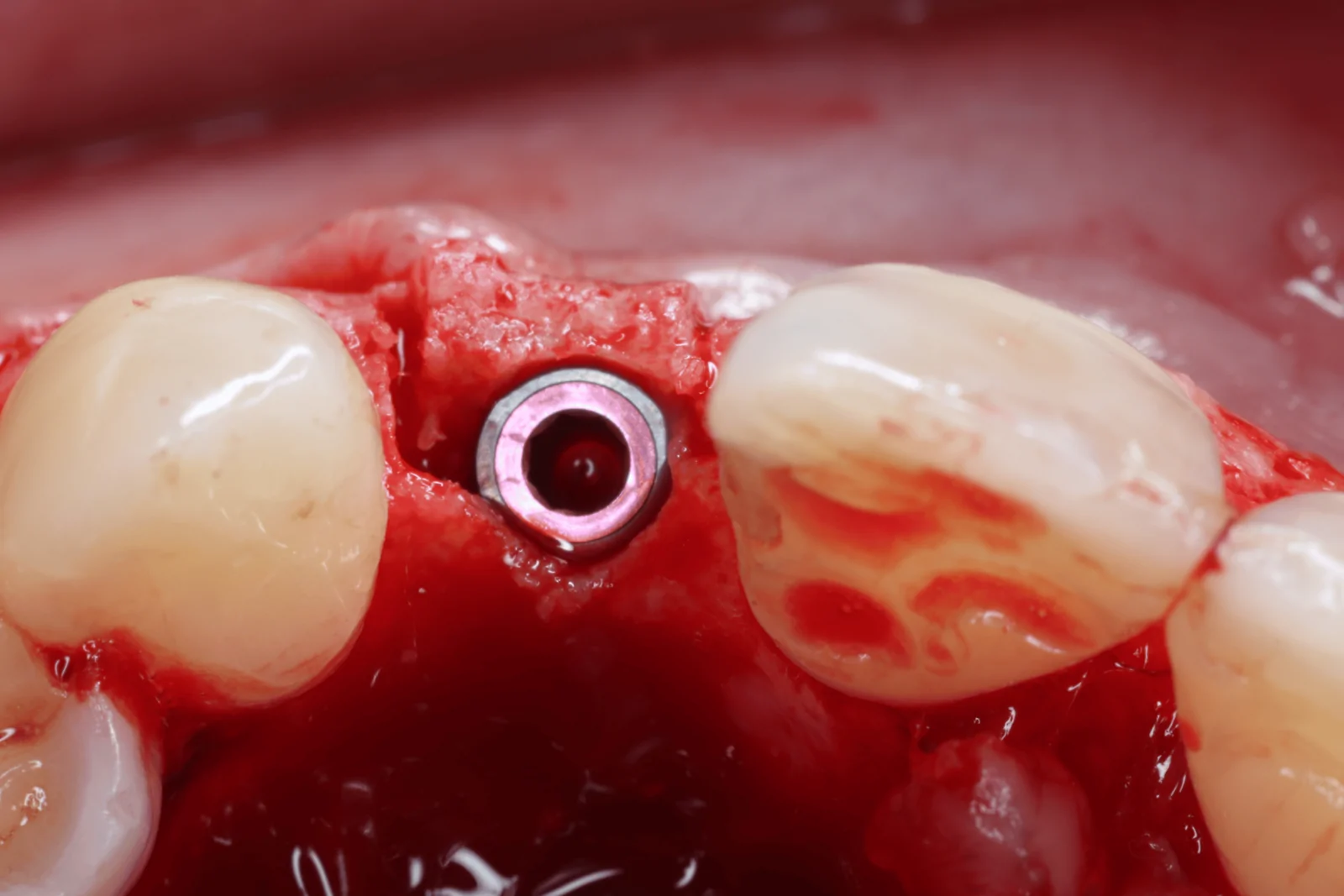
Implant placement following controlled ridge expansion. The implant was positioned prosthetically within the expanded ridge, with adequate space maintained for buccal augmentation.

Guided bone regeneration was then performed. A periosteal releasing incision was made to facilitate tension-free flap closure. A membrane template was used to shape the Jason pericardium membrane, which was first stabilized palatally with two titanium pins. The gap between the buccal and palatal plates and the buccal surface was filled with Cerabone xenograft. The membrane was then secured on the buccal side with two additional titanium pins, using a "sausage technique" to stabilize the graft. The flap was closed with 5-0 resorbable sutures using horizontal mattress sutures, and a periapical radiograph was taken immediately after suturing (Figure 4).

**Figure 4:**
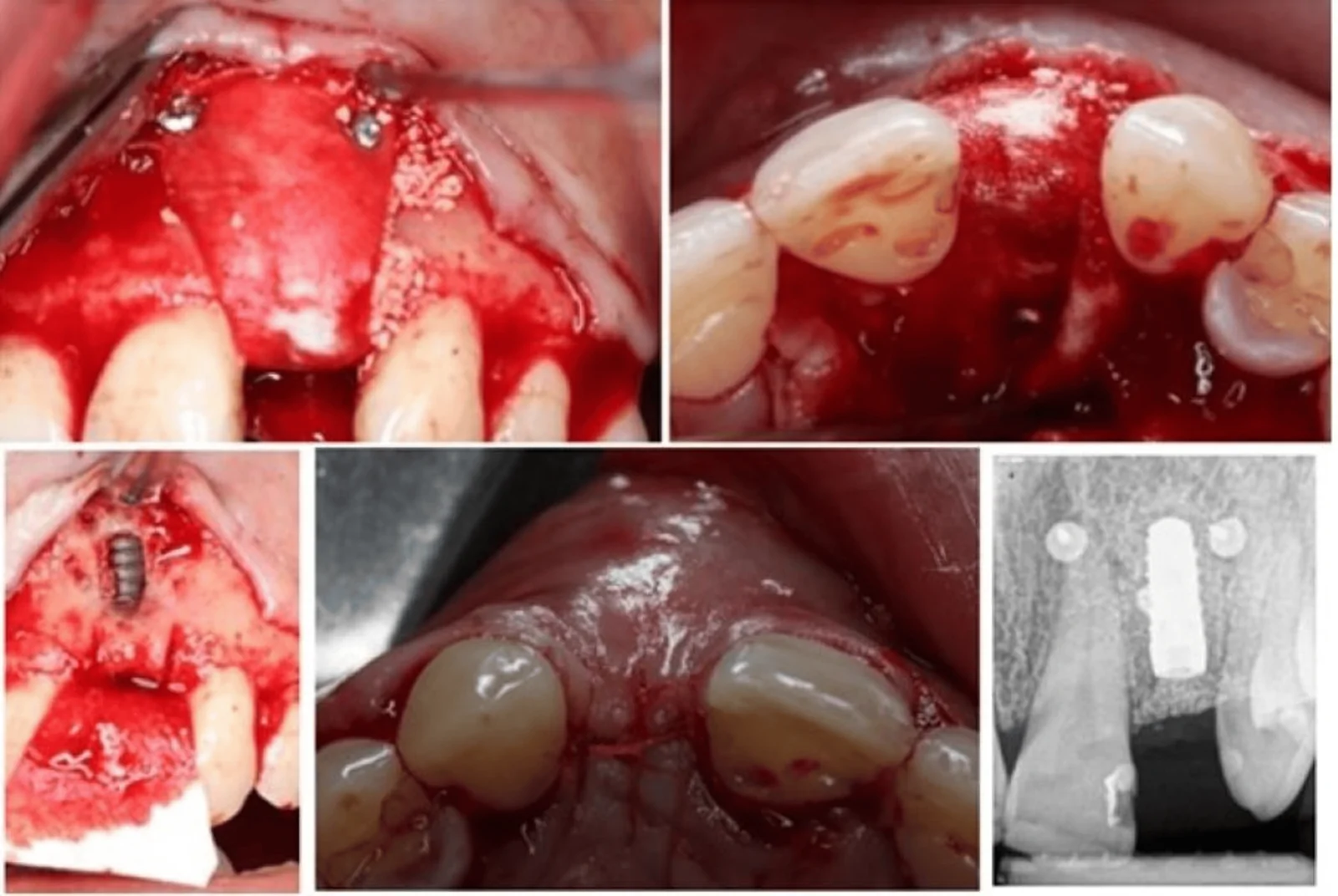
Guided bone regeneration after ridge splitting and implant placement. The split gap and buccal surface were grafted with xenogeneic bone substitute and covered with a pericardium membrane stabilized with titanium pins before tension-free flap closure.

Postoperative care and follow-up

Patients were prescribed amoxicillin-clavulanic acid 1 g twice daily for seven days, paracetamol 500 mg (two tablets every six hours), ibuprofen 200 mg (two tablets every eight hours), and chlorhexidine 0.2% mouthwash twice daily starting 24 hours postoperatively for seven days. They were recalled one week after surgery for clinical evaluation and suture inspection (Figure 5).

**Figure 5:**
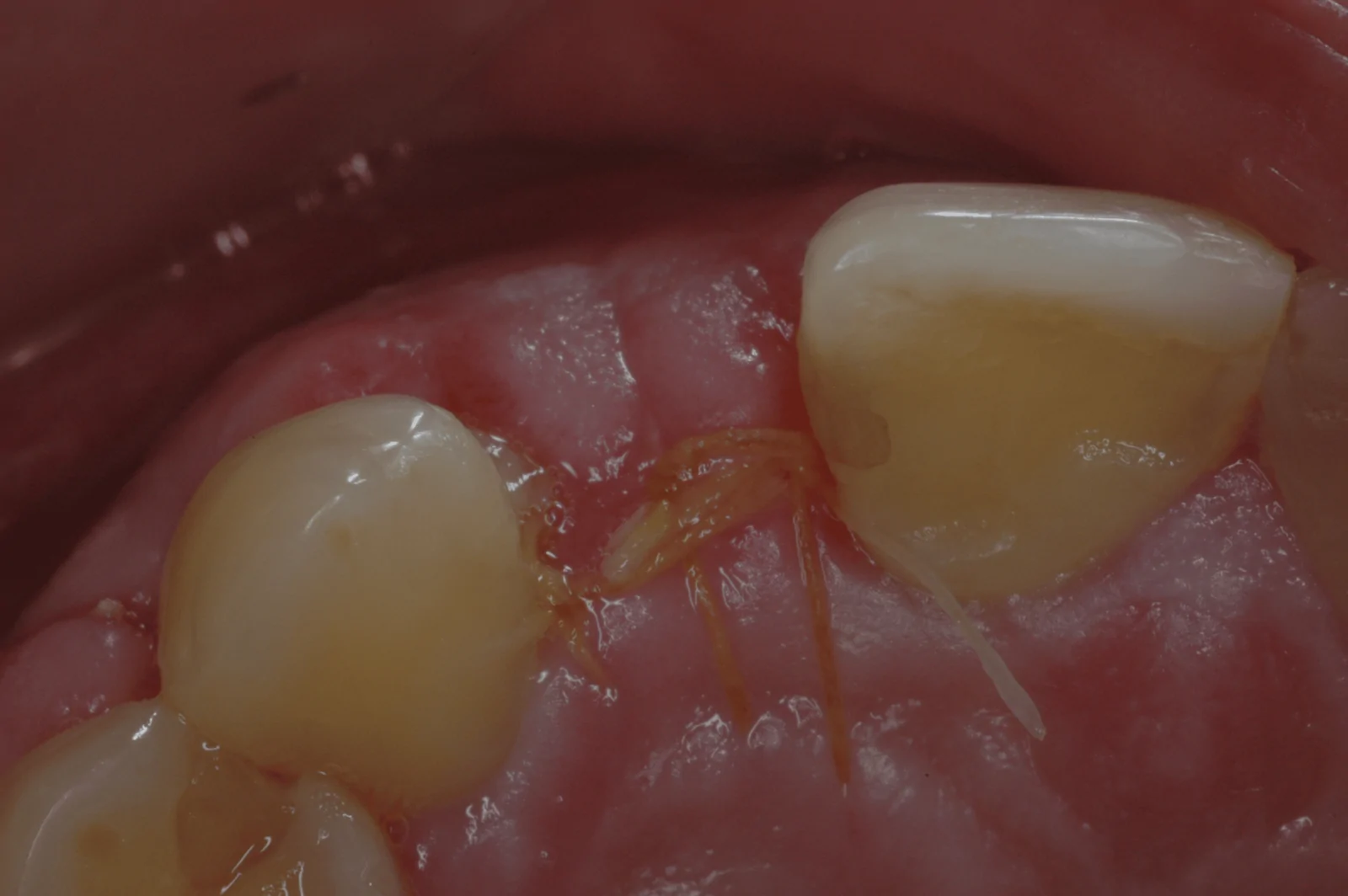
Early postoperative clinical evaluation at one week, showing satisfactory soft-tissue healing and absence of wound dehiscence or membrane exposure.

Sixteen weeks postoperatively, second-stage surgery was performed to uncover the implants and place screw-retained provisional crowns, except in one case where a healing abutment and apically repositioned flap were used due to limited keratinized mucosa (Figure 6).

**Figure 6:**
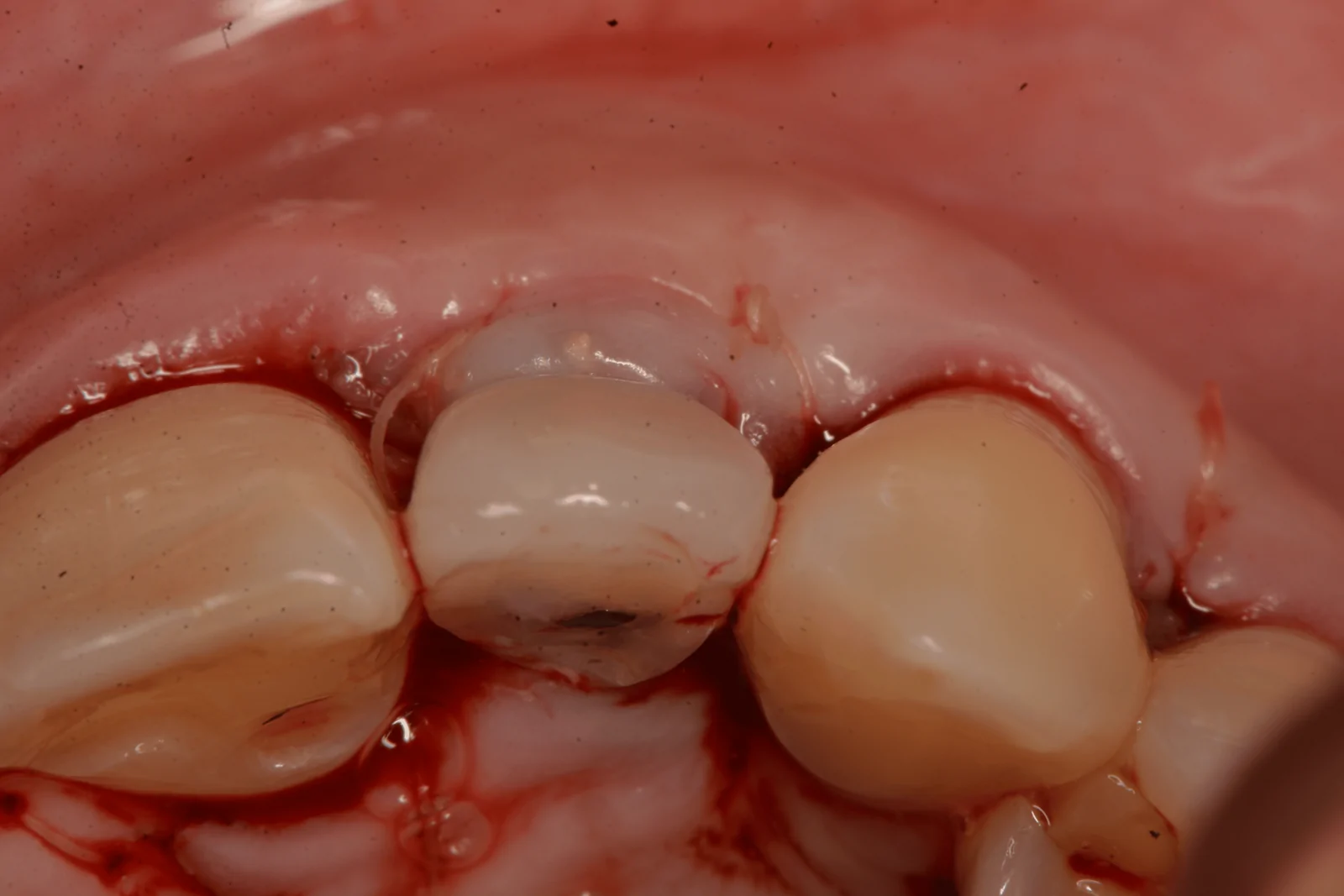
Second-stage surgery at 16 weeks after implant placement. The implant was uncovered for provisionalization; in Case 3, a healing abutment and apically repositioned flap were used because of limited keratinized mucosa.

One month later, definitive impressions were taken and final crowns were delivered (Figure 7).

**Figure 7:**
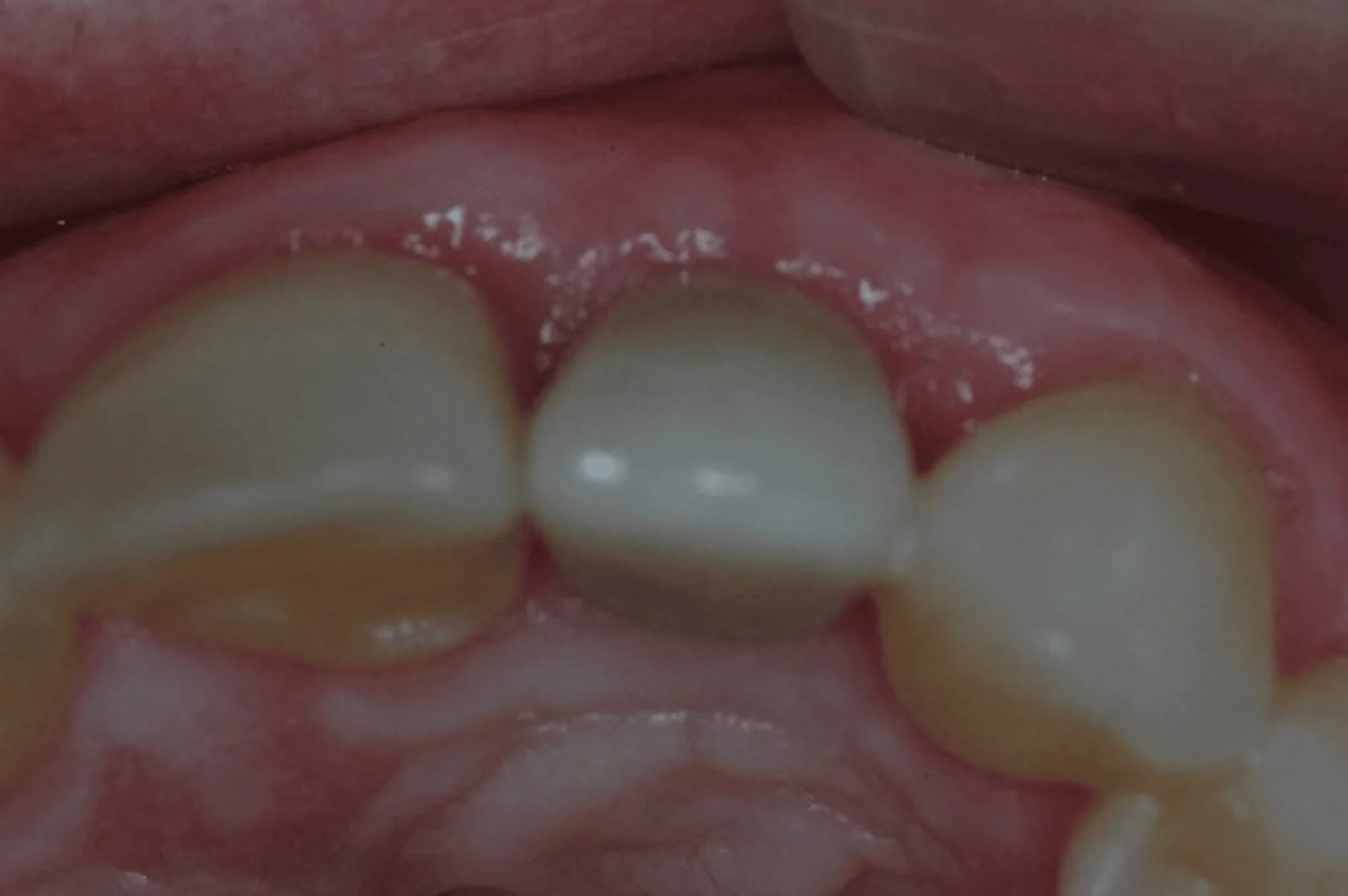
Definitive prosthetic rehabilitation one month after second-stage surgery, showing the final implant-supported crown and peri-implant soft-tissue condition.

Patients were recalled one year after loading for clinical and radiographic evaluation, including periapical radiographs and CBCT scans to assess hard and soft tissue stability (Figure 8).

**Figure 8:**
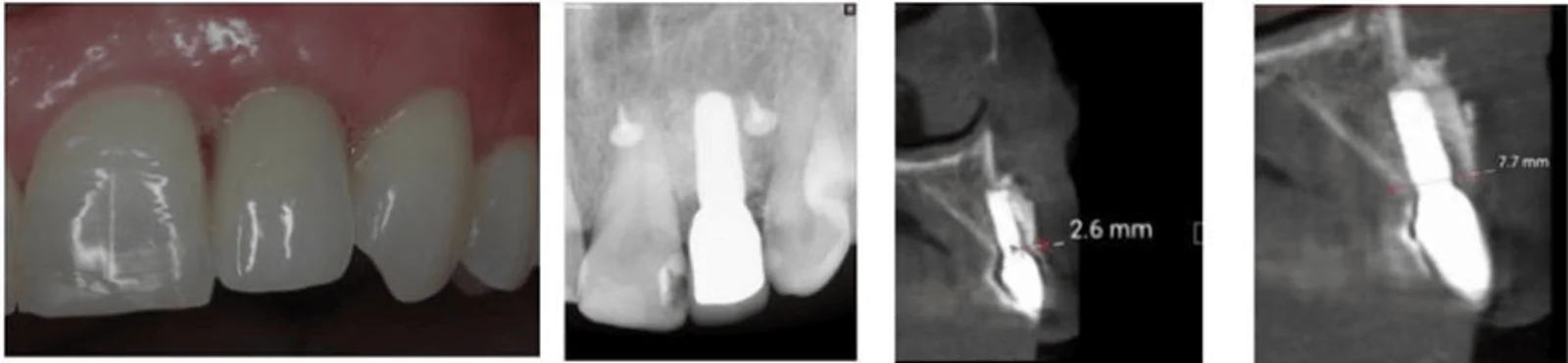
One-year post-loading clinical and radiographic follow-up. The clinical view and radiographs demonstrate implant function and maintenance of the augmented ridge and peri-implant hard and soft tissues.

Case series

All three cases followed the same standardized treatment sequence: ARST using piezosurgery and sequential chisels, simultaneous placement of Klockner implants in a prosthetically driven position, guided bone regeneration using Cerabone xenograft and a Jason pericardium membrane stabilized with titanium pins, second-stage surgery 16 weeks after implant placement, and delivery of definitive crowns one month later. Clinical and CBCT ridge-width measurements were obtained at standardized time points. The principal case-specific variation was the management of limited keratinized mucosa during second-stage surgery in Case 3, in which a healing abutment and apically repositioned flap were used.

Case 1

A 45-year-old woman, light smoker (five cigarettes/day), with no relevant systemic disease and no presence or history of periodontal disease, presented with a healed edentulous site at tooth 14 in the maxillary anterior region. The site had been edentulous for five years. Clinical examination showed 4.0 mm of keratinized mucosa and healthy soft tissue. CBCT demonstrated a horizontal ridge width of 4.0 mm and a residual vertical bone height of ≥10 mm.

ARST with simultaneous implant placement and guided bone regeneration was performed using Cerabone xenograft and a Jason pericardium membrane. A Klockner implant measuring 4.0 × 10 mm was placed with an insertion torque of 25 Ncm. The radiographic ridge width increased to 10.8 mm immediately after surgery, decreased to 7.3 mm at crown delivery, and was 7.8 mm one year after functional loading, resulting in a net radiographic horizontal gain of 3.8 mm. Clinical ridge width increased from 3.5 mm at baseline to 7.0 mm at second-stage surgery, corresponding to a clinical gain of 3.5 mm. A cement-retained definitive zirconia crown was delivered. No intraoperative or postoperative adverse events, membrane exposure, implant mobility, or implant loss occurred during the one-year follow-up.

Case 2

A 50-year-old woman, nonsmoker, with no relevant systemic disease and no evidence of periodontal disease, presented with a healed edentulous site at tooth 22 in the maxillary anterior region. The site had been edentulous for three years. Clinical examination showed 3 mm of keratinized mucosa and intact soft tissues without clinical signs of inflammation. CBCT demonstrated a horizontal ridge width of 4.8 mm and a residual vertical bone height of at least 10 mm.

Following ARST, simultaneous implant placement, and guided bone regeneration using Cerabone xenograft and a Jason pericardium membrane, a Klockner implant measuring 3.5 × 10 mm was placed with an insertion torque of 20 Ncm. The radiographic ridge width increased to 7.5 mm immediately after surgery, 7.6 mm at crown delivery, and 7.7 mm at one year after functional loading, resulting in a net radiographic horizontal gain of 2.9 mm. The small increase in CBCT-measured ridge width from 7.5 mm immediately postoperatively to 7.7 mm at one year after loading was considered a minor variation in serial radiographic measurements and/or local bone remodeling, rather than additional augmentation. Clinical ridge width increased from 4.0 mm at baseline to 7.5 mm at second-stage surgery, corresponding to a clinical gain of 3.5 mm. A cement-retained definitive zirconia crown was delivered. No intraoperative or postoperative adverse events, membrane exposure, implant mobility, or implant loss occurred during the one-year follow-up.

Case 3

A 52-year-old woman, nonsmoker, with no relevant systemic disease and healthy periodontium, presented with posterior maxillary edentulism at tooth 26. The site had been edentulous for seven years. Clinical examination revealed 3.5 mm of keratinized mucosa, with no clinical signs of soft-tissue inflammation. CBCT demonstrated a horizontal ridge width of 4.2 mm and a residual vertical bone height of 13 mm.

ARST with simultaneous implant placement and guided bone regeneration was performed using Cerabone xenograft and a Jason pericardium membrane. A Klockner implant measuring 4.0 × 10 mm was placed with an insertion torque of 30 Ncm. The radiographic ridge width increased to 8.8 mm immediately after surgery and to 9.0 mm at one year after functional loading, resulting in a net radiographic horizontal gain of 4.8 mm. Clinical ridge width increased from 3.5 mm at baseline to 9.0 mm at second-stage surgery, corresponding to a clinical gain of 5.5 mm. At second-stage surgery, a healing abutment and an apically repositioned flap were used because of limited keratinized mucosa. A cement-retained definitive zirconia crown was subsequently delivered. No intraoperative or postoperative adverse events, membrane exposure, implant mobility, or implant loss occurred during the one-year follow-up.

Results

Three patients met the inclusion criteria and completed the planned surgical and prosthetic protocol. Given the descriptive nature of this three-patient case series, no inferential statistical analyses were performed. Individual clinical and radiographic ridge-width measurements at each assessment time point are presented in Table 1 and Table 2. Clinically, all cases demonstrated sufficient horizontal augmentation to maintain at least 2 mm of buccal bone around the implants. Radiographic CBCT measurements demonstrated a mean horizontal bone gain of 3.83 mm (range, 2.9-4.8 mm) at one year after loading. Clinical measurements demonstrated a mean ridge-width increase of 4.16 mm (range, 3.5-5.5 mm) at second-stage surgery. No implant loss, major complications, or membrane exposures were recorded during the follow-up period.

**Table 1:** CBCT-based buccolingual horizontal ridge-width measurements (mm) at the planned implant site before surgery, immediately after surgery, at crown delivery, and one year after functional loading. Ridge width was measured as the buccolingual distance between the outer surfaces of the buccal and palatal cortical plates at the corresponding implant site on cross-sectional images oriented perpendicular to the alveolar crest. Radiographic gain was calculated as the CBCT ridge width at one year after functional loading minus the preoperative CBCT ridge width. CBCT: cone-beam computed tomography.

Case no.	Preoperatively	Postoperatively	Post-loading	One-year post-loading	Gain
Case 1	4.0 mm	10.8 mm	7.3 mm	7.8 mm	3.8 mm
Case 2	4.8 mm	7.5 mm	7.6 mm	7.7 mm	2.9 mm
Case 3	4.2 mm	8.8 mm	8.8 mm	9.0 mm	4.8 mm

**Table 2:** Clinical buccolingual horizontal ridge-width measurements (mm) at the planned implant site before surgery, immediately after surgery, and at second-stage surgery. Clinical ridge width was measured at the corresponding implant site using a North Carolina periodontal probe positioned perpendicular to the alveolar ridge. Clinical gain was calculated as the clinical ridge width at second-stage surgery minus the preoperative clinical ridge width.

Case no.	Preoperatively	Postoperatively	Second stage	Gain
Case 1	3.5 mm	10.0 mm	7.0 mm	3.5 mm
Case2	4.0 mm	7.5 mm	7.5 mm	3.5 mm
Case 3	3.5 mm	9.0 mm	9.0 mm	5.5 mm

Radiographic measurements from CBCT images showed a mean horizontal bone gain of 3.83 mm (range, 2.9-4.8 mm) at one year post-loading (Table 1).

Clinically, the mean increase in ridge width was 4.16 mm, with gains of 3.5 mm, 3.5 mm, and 5.5 mm for Cases 1, 2, and 3, respectively (Table 2).

No implant loss, major complications, or membrane exposures were recorded during the follow-up period.

## Discussion

Augmentation of narrow alveolar ridges is often required to allow prosthetically driven implant placement and long-term success. Simion et al. described the ridge splitting procedure as a "self-space making" defect, suggesting that this defect can effectively contain graft material and favor new bone formation when compared with conventional GBR alone [9]. Cortellini et al. further characterized the defect site as a favorable environment for new vital bone formation [13]. In the present series, ARST combined with simultaneous implant placement and xenograft plus pericardium membrane achieved mean radiographic horizontal gains of 3.83 mm and clinical gains of 4.16 mm, which are comparable to or slightly higher than values reported in previous studies.

Elnayef et al. conducted a systematic review to assess the predictability of successfully performing the ridge split technique for augmentation based on the PICO question: "Can patients with horizontal ridge deficiency be successfully treated with alveolar ridge split technique and implant therapy?" Their results showed an implant survival rate of 97% and a weighted mean horizontal bone gain of 3.19 ± 1.19 mm, concluding that the split technique may be predictable in terms of high implant survival rate, adequate horizontal bone gain, and minimal intraoperative and postoperative complications [14]. Our findings align with these data, further supporting the predictability of this approach.

Abou Hamdan et al. reported a case treated with alveolar ridge splitting and simultaneous implant placement in which a horizontal bone gain of 3.7 mm was maintained after one year of functional loading, despite the absence of graft material within the split gap [6]. This outcome is comparable with the mean radiographic horizontal gain of 3.83 mm observed in the present case series. In our patients, ridge width remained stable or increased slightly between crown delivery and the one-year post-loading assessment, and at least 2 mm of buccal bone was maintained around all implants. Collectively, these findings support the short-term clinical and radiographic stability of the augmented ridge following ARST with simultaneous implant placement and GBR.

Flieger reported a 2019 case report of bilateral maxillary ridge splitting with immediate placement of two implants in a patient with osteoporosis receiving bisphosphonate therapy [15]. The preoperative ridge width was 3-4 mm. At the two-year post-loading follow-up, no marginal bone loss was observed around the implant collars. Although this single case suggests a favorable outcome, it does not establish the predictability of ridge splitting in medically compromised patients.

Kumar et al. evaluated soft- and hard-tissue changes following the ARST in 2019. The initial ridge width was mm, with a mean horizontal gain of mm [8]. These findings are broadly consistent with the horizontal augmentation observed in the present case series, although comparisons should be interpreted cautiously because of differences in study design, surgical protocol, grafting approach, and measurement methods.

Abou Hamdan and Salame performed a study on 160 mandibular and maxillary implant sites, of which 140 implants were placed immediately during the alveolar ridge split procedure [16]. Their results showed a success rate of 97.5% according to Buser's criteria, demonstrating the technique's reliability across a larger sample.

Yadav et al. performed a clinical trial on 22 patients and assessed implant stability and bone width six months following the ridge split surgery for horizontal bone augmentation [17]. The results showed an implant stability of 86.36% and a bone width that augmented from 3.1-4.4 mm initially to 5.2-6.3 mm six months postoperatively, demonstrating that this technique is predictable for horizontal bone augmentation. However, this study showed a mean bone gain of 2.0 ± 0.1 mm, whereas our results demonstrated a mean gain of 3.83 mm, possibly due to differences in surgical technique, graft materials, or measurement protocols.

The higher bone gain observed in Case 3 may be related to the posterior edentulous site, where a broader defect allowed more extensive augmentation and potentially greater blood supply from the surrounding bone. In addition, all procedures were performed by the same experienced surgeon using the same implant system and biomaterials, which may have contributed to the consistency and favorable outcomes observed.

Patients receiving antiplatelet therapy were not represented in this small case series; therefore, the findings cannot be directly extrapolated to this population. In clinical practice, implant surgery in patients with cardiovascular disease receiving antiplatelet therapy requires an individualized assessment of cardiovascular stability, the indication for antiplatelet therapy, thrombotic risk, and the anticipated surgical bleeding risk, with consultation with the treating physician or cardiologist when indicated. Antiplatelet medication should not be interrupted or modified without the recommendation of the prescribing physician, because discontinuation may expose patients to serious thrombotic complications. When surgery is considered appropriate, a minimally traumatic approach, careful flap management, meticulous local hemostasis, suturing, and close postoperative surveillance are advised.

This case series is limited by the small sample size of three patients and the relatively short follow-up period of one year after loading. Because of the descriptive design and the very small sample size, no inferential statistical analysis was undertaken, and the findings should be interpreted as exploratory case-level observations rather than estimates of treatment efficacy or generalizable clinical outcomes. Measurements were performed by a single calibrated examiner; however, repeat measurements, formal intra-examiner reliability testing, and blinding to assessment time point were not undertaken, which may have introduced measurement bias. Although the present findings indicate favorable clinical and radiographic ridge stability at one year, this observation is insufficient to establish the long-term dimensional stability of the augmented ridge. In addition, the findings cannot be extrapolated to patients previously treated with head-and-neck radiotherapy, as radiation-associated alterations in bone vascularity and remodeling may compromise wound healing, implant osseointegration, and regenerative outcomes. Similarly, the present findings cannot be directly generalized to patients with cardiovascular disease receiving antiplatelet therapy. Larger prospective studies with longer follow-up periods and standardized three-dimensional radiographic measurements are needed to assess ridge remodeling over time, confirm the long-term stability of the augmented bone, and evaluate the survival of implants placed using this technique. Future investigations should also incorporate patient-reported outcome measures and comparative analyses with alternative horizontal ridge augmentation approaches.

## Conclusions

Within the limitations of this three-patient case series, ARST with simultaneous implant placement and guided bone regeneration using xenogeneic graft material and a pericardium membrane was feasible in carefully selected narrow maxillary ridges. All cases demonstrated horizontal ridge augmentation, with a mean radiographic bone gain of 3.83 mm and a mean clinical gain of 4.16 mm. Adequate buccal bone thickness and stable clinical and radiographic conditions were maintained at one year after functional loading, with no implant loss or major complications observed.

These findings indicate favorable short-term outcomes and support the potential of ARST as a treatment option for selected maxillary horizontal ridge deficiencies. However, owing to the small sample size, absence of a comparator group, selective case inclusion, and limited one-year follow-up, these results should not be interpreted as evidence of broad clinical predictability or generalizability. Larger prospective comparative studies with longer follow-up are needed to confirm long-term stability, define appropriate case-selection criteria, and evaluate ARST against alternative ridge-augmentation approaches.
